# Synergistic Enhancement of Facial Nerve Regeneration Using a Cellulose-Based Nerve Conduit Loaded with Mesenchymal Stem Cells and Human Placental Extract

**DOI:** 10.3390/bioengineering13080935

**Published:** 2026-08-18

**Authors:** Chul Ho Jang, Gwang-Won Cho, Gyeong Min Im, Minseong Kim

**Affiliations:** 1 Department of Otolaryngology, Gwangju Veterans Hospital, Gwangju 62284, Republic of Korea; 2 Department of Otolaryngology, Chonnam National University Medical School, Gwangju 61469, Republic of Korea; 3 Department of Life Science, BK21-Plus Research Team for Bioactive Control Technology, Chosun University, Gwangju 61452, Republic of Korea; gwcho@chosun.ac.kr (G.-W.C.); a49463640@gmail.com (G.M.I.); 4 Medical Device Development Center, KBIO Health Osong Medical Innovation Foundation, Cheongju-si 28160, Republic of Korea; mkim@kbiohealth.kr

**Keywords:** facial nerve, regeneration, nerve conduit, cellulose nanofiber, mesenchymal stem cell, placental extract

## Abstract

**Background**: Facial nerve regeneration across segmental defects remains challenging despite advances in nerve guidance conduits. Tissue engineering strategies that combine biomaterial scaffolds with bioactive and cellular components may enhance functional and structural nerve repair. This study investigated the regenerative efficacy of a cellulose-based nerve conduit augmented with Matrigel, mesenchymal stem cells (MSCs), and placental extract (PE) in a rat facial nerve gap model. **Methods**: A 2 mm defect was created at the main trunk of the facial nerve in adult Sprague–Dawley rats. Animals were divided into three groups: control group (Cellulose/Matrigel), group I (Cellulose/Matrigel/MSC), and group II (Cellulose/Matrigel/MSC/PE). Functional recovery was assessed at 2, 4, 6, and 8 weeks postoperatively using slow-motion vibrissa movement analysis, electrically stimulated facial nerve action potential measurements, and facial nerve blood flow by laser Doppler blood flow analysis. Morphological regeneration was evaluated using light microscopy and transmission electron microscopy. Western blotting using facial muscle was performed. **Results**: All groups exhibited time-dependent facial nerve regeneration. However, group II demonstrated significantly enhanced functional recovery, greater electrophysiological responses, increased nerve blood flow, and superior histological and ultrastructural regeneration compared with the other groups. These improvements were particularly prominent at 6 and 8 weeks postoperatively. **Conclusions**: The combination of mesenchymal stem cells and PE within a cellulose-based nerve conduit synergistically enhanced facial nerve regeneration. This tissue-engineered strategy may represent an effective approach for improving peripheral nerve repair.

## 1. Introduction

The facial nerve is the peripheral nerve most frequently affected by iatrogenic, traumatic, and idiopathic injury, and its dysfunction imposes a heavy burden on patients because facial expression underlies both social communication and emotional well-being [1,2]. Iatrogenic injury during parotidectomy, mastoidectomy, and temporomandibular joint surgery, together with temporal-bone fractures and tumor resection, accounts for the majority of clinically significant facial nerve lesions, and most of these injuries are graded using the House–Brackmann scale [1,3]. When continuity of the nerve cannot be restored by primary coaptation, autologous nerve grafting is considered the gold standard, but donor-site morbidity, limited graft length, size mismatch, and incomplete functional recovery remain substantial problems [2,4,5].

Tissue-engineered nerve guidance conduits (NGCs) have therefore attracted intense interest as an alternative to autografts for bridging peripheral nerve defects [4,6]. Among candidate biomaterials, cellulose and its derivatives are particularly attractive because of their biocompatibility, tunable mechanical properties, and ability to be processed into aligned nanofibers that guide directional axonal outgrowth and Schwann cell migration [6,7]. Biosynthetic cellulose conduits have produced nerve-regeneration outcomes approaching those of autografts when combined with cellular or molecular cargo [7,8]. Our group has previously shown that cellulose/collagen nanofibrous conduits coated with pre-induced mesenchymal stem cells support regeneration of a transected rat facial nerve [9].

A hollow conduit alone, however, rarely provides sufficient biological cues to overcome longer or more complex defects [6]. Supplementation with Schwann cells or Schwann-like cells has long been recognized as beneficial, but the limited availability and donor morbidity of autologous Schwann cells have driven a shift toward mesenchymal stem cells (MSCs) as a more accessible cellular component [10]. MSCs promote peripheral nerve regeneration chiefly through paracrine secretion of neurotrophic factors—including brain-derived neurotrophic factor (BDNF), nerve growth factor (NGF), and neurotrophin-3—and through transdifferentiation into Schwann-like cells that participate in remyelination [11]. The extracellular-matrix component of the construct is equally important. Matrigel, a laminin- and collagen IV-rich basement-membrane matrix, markedly enhances the survival, adhesion, and axon-promoting activity of transplanted Schwann and Schwann-like cells and has become a standard carrier for cell delivery in peripheral nerve regeneration studies [12]. Coupling Matrigel with a structural cellulose conduit yields a composite in which macroscopic guidance is provided by the scaffold and the microenvironmental cues for seeded cells are supplied by the hydrogel.

Human placental extract (PE) is a clinically used preparation rich in growth factors such as EGF, FGF, HGF, IGF, PDGF, TGF, VEGF, and NGF, together with amino acids, peptides, and anti-inflammatory cytokines [13,14]. PE supports Schwann cell proliferation and migration in vitro, upregulates the expression of neurotrophic genes, and accelerates functional and morphological recovery in a rat facial nerve crush injury model [14]. Repeated epidural injection of human placental extract in a lumbar spinal stenosis model likewise enhanced axonal plasticity and reduced neuroinflammation, underscoring its neuroregenerative potential [13]. Because a single recombinant growth factor is unlikely to recapitulate the complex trophic environment required for nerve regeneration, the broad spectrum of native factors contained in PE is particularly attractive for use in a nerve-conduit context.

Despite these advances, no previous study has combined a cellulose nanofiber conduit with a Matrigel/MSC/PE filling in a facial nerve transection model. The present study was designed to fill that gap. We hypothesized that delivering MSCs together with placental extract inside a cellulose/Matrigel conduit would provide cellular, matricellular, and trophic support in a single construct and would therefore produce superior axonal regeneration and remyelination compared with either the unfilled conduit or the conduit with MSCs alone. We tested this hypothesis using in vitro and in vivo approaches using functional, morphological and molecular measurements.

## 2. Materials and Methods

### 2.1. Cell Culture

Human bone marrow mesenchymal stem cells (hBM-MSCs) were obtained and maintained as previously described [15]. The cells were continuously observed using a bright-field microscope (Nikon Eclipse TS100; Tokyo, Japan) and subcultured upon reaching confluence. Briefly, from Cell Engineering for Origin (Seoul, Korea), hBM-MSCs were acquired. It was verified that the cells were free of mycoplasma, viruses, and bacteria. At 37 °C and 5% CO_2_, cells were cultivated in low-glucose Dulbecco’s modified Eagle’s medium (DMEM) mixed with 10% fetal bovine serum (FBS), L-glutamine, and 1% penicillin/streptomycin. Experiments involving high glucose were conducted at 37 °C and 5% CO_2_ in high-glucose DMEM supplemented with 10% FBS, L-glutamine, and 1% penicillin/streptomycin. Subculturing was carried out when the cells achieved confluence, as observed by a bright-field microscope (Nikon Eclipse TS100, Tokyo, Japan), with the media being replaced every three days.

### 2.2. Cell Viability Assay

To assess the proliferative effects of placental extract (PE) on hBM-MSCs, an MTT assay (Sigma, St Louise, MO, USA) was performed following the manufacturer’s protocol. The cells were seeded at a density of 8 × 10^3^ cells per well in 96-well plates. After 24 h, they were treated with PE at concentrations ranging from 0 to 5 mg/mL for an additional 24 h before performing the MTT assay.

### 2.3. Cell Migration Assay

For the cell migration study, hBM-MSCs were plated in 12-well plates at a density of 5 × 10^4^ cells per well and incubated at 37 °C for 24 h. After reaching 80% confluence, the cells were exposed to PE for 24 h at 37 °C. A sterile P200 pipette tip was used to create a scratch approximately 1.0 mm wide in the cell monolayer. ImageJ (1.54t; 16 May 2026) software (https://imagej.nih.gov) was utilized to measure the area covered by migrating cells.

### 2.4. Oxidative Stress-Induced Senescence

Oxidative stress-induced cell cycle arrest and senescence were induced using hydrogen peroxide (H_2_O_2_), following established methods [16]. The cells were treated with 200 µM H_2_O_2_ for 2 h and then cultured for 2 days in standard media. For a subsequent treatment, the cells were split at a 1:3 ratio, re-exposed to 200 µM H_2_O_2_ for 2 h, and cultured for 72 h in either normal media (control), PE (2 mg/mL), or ascorbic acid (500 µM, a positive control).

### 2.5. Senescence-Associated β-Galactosidase (SA-β-Gal) Staining

Cellular senescence was evaluated using an SA-β-Gal assay kit as described previously [15]. Following 72 h of treatment, the cells were washed with phosphate-buffered saline (1X PBS; Takara Bio Inc., Kusatsu, Shiga, Japan) and fixed with 1X fixative for 10–15 min at room temperature. The SA-β-Gal staining solution (pH 6.0) was added, and the plates were sealed with parafilm and incubated overnight in a dry, CO_2_-free incubator at 37 °C. SA-β-Gal-positive cells were visualized under a light microscope (Nikon Corporation, Eclipse TS100; Tokyo, Japan) and images were captured using an IMTcam3 camera (IMT i-Solution Inc., Brook Anco Corporation, Cicero, NY, USA).

#### 2.5.1. Fabrication of Nerve Conduit

**Figure 1:**
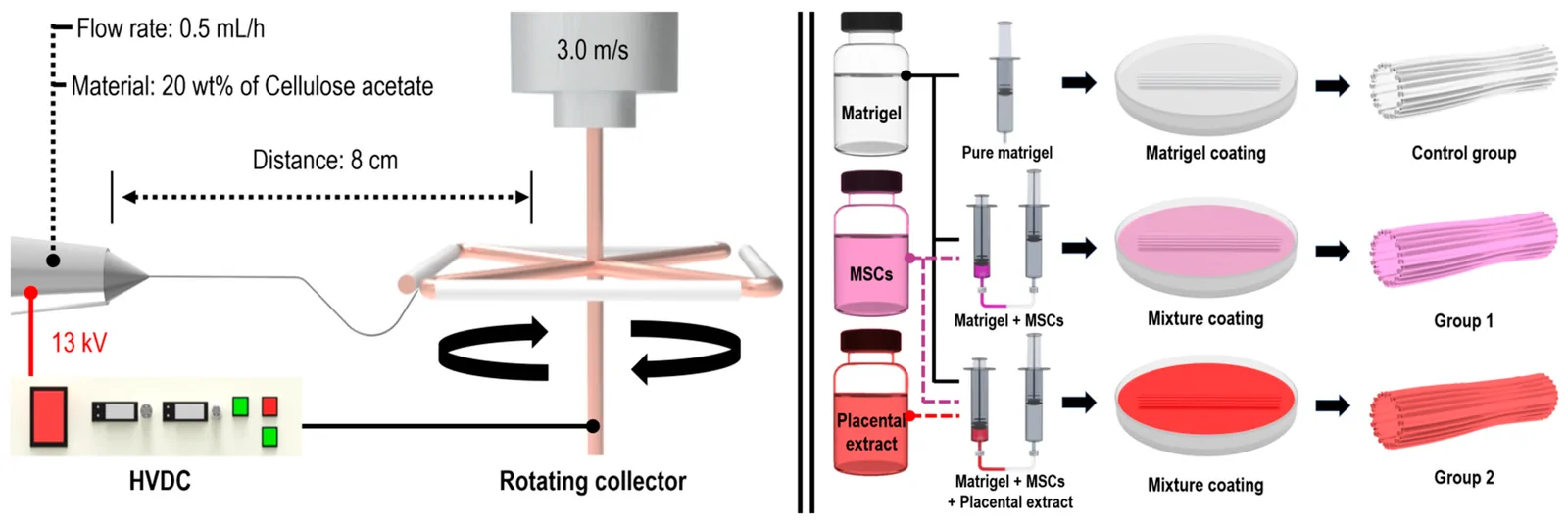
**Fabrication of cellulose-based nerve conduit and experimental group preparation**. (**Left**) Schematic illustration of the electrospinning setup used to fabricate the conduit, showing a high-voltage power supply (13 kV), flow rate (0.5 mL/h), cellulose acetate solution (20 wt%), tip-to-collector distance (8 cm), and a rotating collector (3.0 m/s). (**Right**) Preparation of experimental groups. The control group received the Matrigel coating only; Group 1 received a mixture of Matrigel and mesenchymal stem cells (MSCs); and Group 2 received a combination of Matrigel, MSCs, and placental extract. Coated conduits were then prepared for implantation.

A cylindrical cellulose scaffold was fabricated using an electrospinning technique. Cellulose acetate (density: 1.3 g/cm^3^, Mn: 30,000 g/mol) was obtained from Sigma-Aldrich (USA) for electrospinning applications. A 20 wt% cellulose solution was prepared using a solvent system consisting of acetone (JUNSEI, Saitama, Japan) and dimethylformamide (JUNSEI, Japan) in a 1:1 ratio. Figure 1 illustrates the electrospinning setup, which consisted of a high-voltage direct current power source, an electrospinning nozzle, and a rotating collector. The cellulose solution was dispensed at a controlled rate of 0.5 mL/h using a syringe pump under an applied voltage of 13 kV. The collector rotated at approximately 0.3 m/s, and the nozzle was positioned 8 cm away from the collector. The electrospinning process was conducted for 2 h. After fiber deposition was complete, the nanofibrous conduit was carefully detached from the rotating collector. Following detachment, the conduit scaffolds were washed with 70% ethanol and dried. The control group of conduits was then immersed in a Matrigel solution (Corning, USA). For group 1, the cellulose conduits were coated with Matrigel and 10^6^ of MSC mixture. To fabricate group 2, Matrigel, 10^6^ cell density of MSCs, and placental extract were mixed and coated onto the nanofibrous cellulose conduit scaffolds. Each group of conduit scaffolds was coated for 1 h at room temperature and then cross-linked in a 37 °C incubator for 1 h (Figure 1).

#### 2.5.2. Preparation of Placental Extract

Placental extract (PE) was prepared following the method reported by Heo et al. [17] with minor modifications. Human placental tissue was obtained from Chosun University Hospital. Collection of the placental tissue was performed with written informed consent and was approved by the Institutional Review Board of Chosun University Hospital (IRB No. CHOSUN 2022-10-024-002). The collected tissue was first rinsed with ice-cold phosphate-buffered saline (PBS) to remove residual blood. It was then cut into small fragments and homogenized in chilled PBS for approximately 10 min using a tissue homogenizer (Biospec Products Inc., Bartlesville, OK, USA). The homogenized samples were centrifuged at 6000× *g* for 20 min at 4 °C. After centrifugation, the supernatant was collected and lyophilized to obtain PE powder. The resulting PE powder was reconstituted in 1× PBS at a concentration of 20 mg/mL and sterilized through a 0.22 µm filter prior to use in subsequent experiments.

### 2.6. Induction of Transection Injury-Induced Facial Nerve Paralysis and Nerve Conduit Treatment

**Figure 2:**
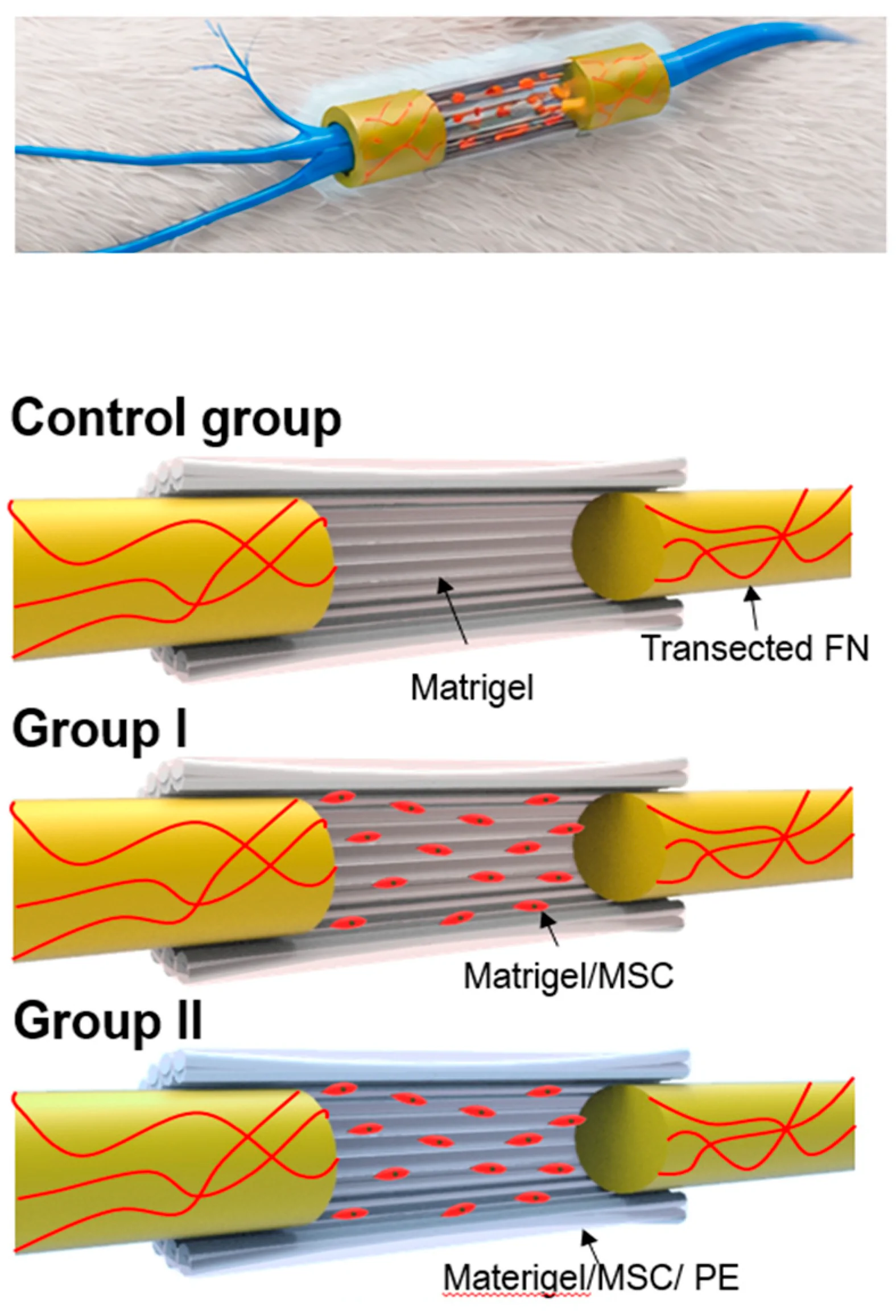
**Schematic and surgical procedure of facial nerve regeneration using a bioengineered nerve conduit**. (**Upper**) Schematic illustration of a 2 mm nerve gap bridged using a tubular conduit. (**Lower**) Diagram of the conduit composition and treatment conditions, including cellulose scaffold, Matrigel, mesenchymal stem cells (MSCs), and placental extract, applied to the transected facial nerve (FN).

Thirty adult male Sprague-Dawley rats (6–8 weeks old, weighing 200–250 g; SamtakoBio Korea, Suwon, Republic of Korea) were used in this study. The rats were randomly divided into three treatment groups (*n* = 10 in each): cellulose nanofiber/Matrigel (control group), cellulose nanofiber/Matrigel/MSCs (group I), and cellulose nanofiber/Matrigel/MSCs/placental extract, 100 μL, 2 mg/mL (group II). Each rat was housed in a separate cage and provided with feed and water. They were allowed to adapt to the environment without stress for a week before surgery. This study was approved by the Animal Experimentation Committee (CIACUC2024-SOOO8). A postauricular incision on the left side was performed because the left side allows easy setup for measuring action potential threshold. After identification of the main trunk of the facial nerve using a surgical microscope (Leica Co., Wetzlar, Germany), a nerve defect was created by transection through a cut in the middle of the main trunk using a microscissor. A 4 mm length cellulose/collagen nanofiber conduit was interposed in this area, and the transected proximal and distal nerve stumps were anchored to the conduit using fibrin glue. A 2 mm gap was thus formed in the main trunk (Figure 2). Finally, the wound was closed using automatic suture. The rats were maintained in a controlled animal breeding facility for 8 weeks after the experiment, where environmental conditions were carefully regulated. The animals were housed under standard laboratory conditions with controlled humidity (approximately 50–60%), minimal ambient noise, and a stable temperature (22–24 °C). They were kept on a 12 h light/dark cycle and provided ad libitum access to standard laboratory chow and water to ensure proper recovery and normal physiological maintenance during the post-experimental breeding period.

### 2.7. Evaluation of Vibrissa Movement Recovery Using Slow Motion Video Analysis Software (BORIS)

Recovery of vibrissa movement was assessed using slow-motion video analysis that is similar to our previous report [14,18,19,20]. During recording, rats were gently restrained in a modified plastic bottle that stabilized the body while allowing the head to remain exposed. In both groups, whisker movements on the left (injured) side were recorded using a high-resolution digital camera (iPhone, Apple Inc., Cupertino, CA, USA) following tactile stimulation using a soft brush at 2, 4, 6, and 8 weeks after transection injury-induced facial nerve paralysis. The recorded videos were analyzed in slow motion, and the number of whisker fibrillations was quantified using Behavioral Observation Research Interactive Software (BORIS), a freely available behavioral analysis program developed by Oliver Friard and Marco Gamba (Department of Life Sciences and Systems Biology, University of Turin, Turin, Italy). Each week, the frequency of vibrissa movement on the injured left side was compared with that of the normal right side, and the results were subsequently analyzed between the control and experimental groups.

### 2.8. Electrically Stimulated Facial Nerve Action Potential Measurement

At 8 weeks after surgery, the facial nerves were surgically re-exposed under general anesthesia with isoflurane inhalation. The nerve segment distal to the crush lesion was stimulated using a monopolar tungsten probe, and the action potential threshold was determined as previously reported [18,19,20]. In brief, the midpoint between the left orbicularis oculi and orbicularis oris muscles was stabilized percutaneously with three paired needle electrodes. A separate needle electrode was placed superficially near the skin as the ground to record electrically evoked muscle action potentials (MAPs). Electrical stimulation was delivered to the main facial nerve trunk using a monopolar stimulating electrode (Xomed-Treace, Jacksonville, FL, USA) connected to a pulse generator, applying rectangular current pulses of 0.05 ms (A-320D; World Precision Instruments Inc., Sarasota, FL, USA). The stimulating probe’s orientation and position relative to the nerve were fine-tuned with a micromanipulator. MAP signals were recorded under maximal stimulation. Data were acquired automatically using a Samsung monitor and the PowerLab/LabChart 8 system (AD Instruments, Castle Hill, Australia) and then analyzed with Scope software (AD Instruments). Recovery after facial nerve injury was quantified by measuring the peak amplitude of the evoked action potential waveform.

### 2.9. Measurement of Facial Nerve Blood Flow Using Laser Doppler Blood Flowmeter

At postoperative week 8, 5 rats from each group were anesthetized via intraperitoneal injection of xylazine hydrochloride combined with tiletamine–zolazepam (Zoletil; Virbac, Carros, France). Using Zoletil with xylazine made the recumbent positioning more practical for measuring facial nerve blood flow (FNBF). The main trunk of the facial nerve was then carefully re-exposed, and the femoral artery was also located. Regional FNBF was measured with a laser Doppler blood flowmeter as previously described [19,20]. For systemic blood pressure (SBP) monitoring, the femoral artery was cannulated and connected to a pressure transducer (AD Instruments, Castle Hill, Sydney, NSW, Australia). A 1.0 mm needle probe was placed on the facial nerve trunk at a perpendicular angle, with care taken to prevent nerve compression, and connected to a laser Doppler flowmeter (moorLAB, Moor Instruments, Axminster, Devon, UK). FNBF and SBP readings were sampled every 20 s and recorded/analyzed using the PowerLab data acquisition system (AD Instruments) with a Samsung laptop (Suwon, Republic of Korea). FNBF was monitored continuously for 30 min.

### 2.10. Assessment of the Regenerated Facial Nerve Using Light Microscopy and Transmission Electron Microscopy

Following euthanasia, distal segments of the facial nerve were promptly harvested from two animals in each group using microsurgical scissors and immediately fixed in 2.5% glutaraldehyde for transmission electron microscopy (TEM). The specimens were rinsed with phosphate buffer and subsequently post-fixed in 1% osmium tetroxide. After graded ethanol dehydration, the tissues were embedded in LR White resin. Transverse semi-thin sections (5 μm) were prepared with a microtome, stained with toluidine blue, and examined under light microscopy. Ultrathin sections were then obtained from the same blocks and analyzed using a JEM-2100F field-emission transmission electron microscope (JEOL Ltd., Tokyo, Japan). Myelin sheath thickness was quantitatively measured using ImageJ (1.54t; 16 May 2026) software.

### 2.11. Immunoblotting

Established procedures were followed to extract and quantify total protein [14]. Radioimmunoprecipitation assay buffer containing protease inhibitors was used to lyse the protein and then the resulting protein extract was centrifuged. Protein concentrations were measured using the bovine serum albumin assay. For Western blotting, specific antibodies for Myogenin (1:500), MyoD (1:500), and GAPDH (1:5000) were used. Secondary antibodies conjugated with horseradish peroxidase and enhanced chemiluminescence staining were then used for detection.

### 2.12. Statistical Analysis

All statistical analyses were conducted using GraphPad Prism version 8.0. Differences among the three groups were evaluated with a one-way analysis of variance (ANOVA). Statistical significance was defined as a *p*-value < 0.05.

## 3. Results

### 3.1. In Vitro Study

#### 3.1.1. Proliferation Effect of Placental Extract in hBM-MSCs

**Figure 3:**
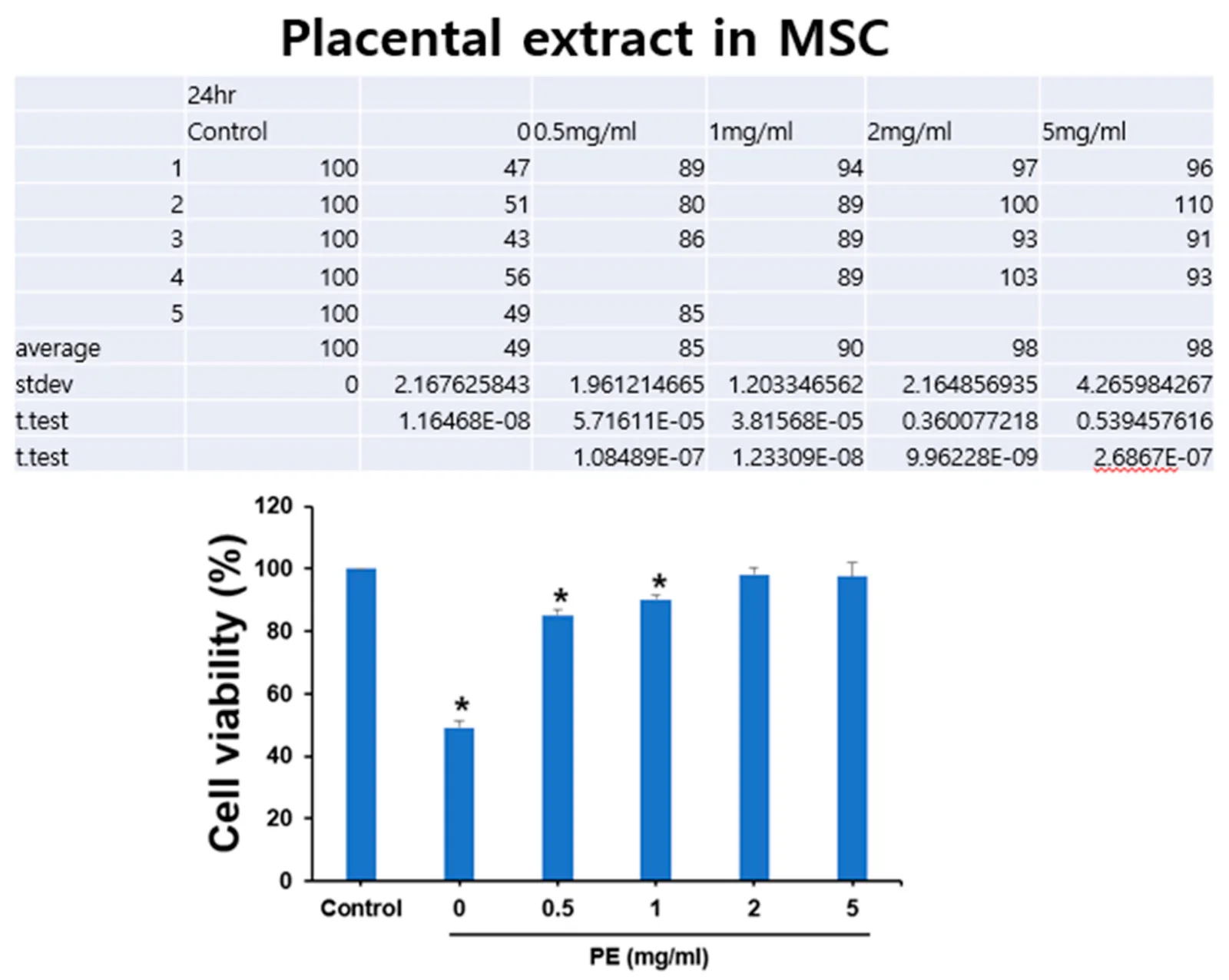
**Effect of placental extract on MSC viability**. (**Top**) Raw data and statistical summary of cell viability (%) in MSCs after 24 h of treatment with placental extract (PE) at different concentrations (0, 0.5, 1, 2, and 5 mg/mL). (**Bottom**) Bar graph showing dose-dependent effects of PE on cell viability. Low-dose PE (0–1 mg/mL) significantly increased cell viability compared to the control, while higher concentrations (2–5 mg/mL) maintained viability near baseline levels. Data are presented as mean ± SEM (* *p* < 0.05 vs. control).

After 24 h of exposure to placental extract (PE), MSC viability showed a concentration-dependent response (Figure 3). The control group maintained 100% viability. Treatment with 0.05 mg/mL PE markedly reduced cell viability to 49 ± 2.17% (*p* < 0.001 vs. control). Increasing the PE concentration significantly improved viability, reaching 85 ± 1.96% at 0.5 mg/mL and 90 ± 1.20% at 1 mg/mL (*p* < 0.001 vs. control). At higher concentrations (2 mg/mL and 5 mg/mL), cell viability recovered to 98 ± 2.16% and 98 ± 4.27%, respectively, with no significant difference compared with the control (*p* = 0.36 and *p* = 0.54). These findings indicate that very low concentrations of PE reduce MSC viability, whereas concentrations ≥ 2 mg/mL maintain viability comparable to untreated controls.

#### 3.1.2. Effect of PE on MSC Migration in the Wound Healing Assay

**Figure 4:**
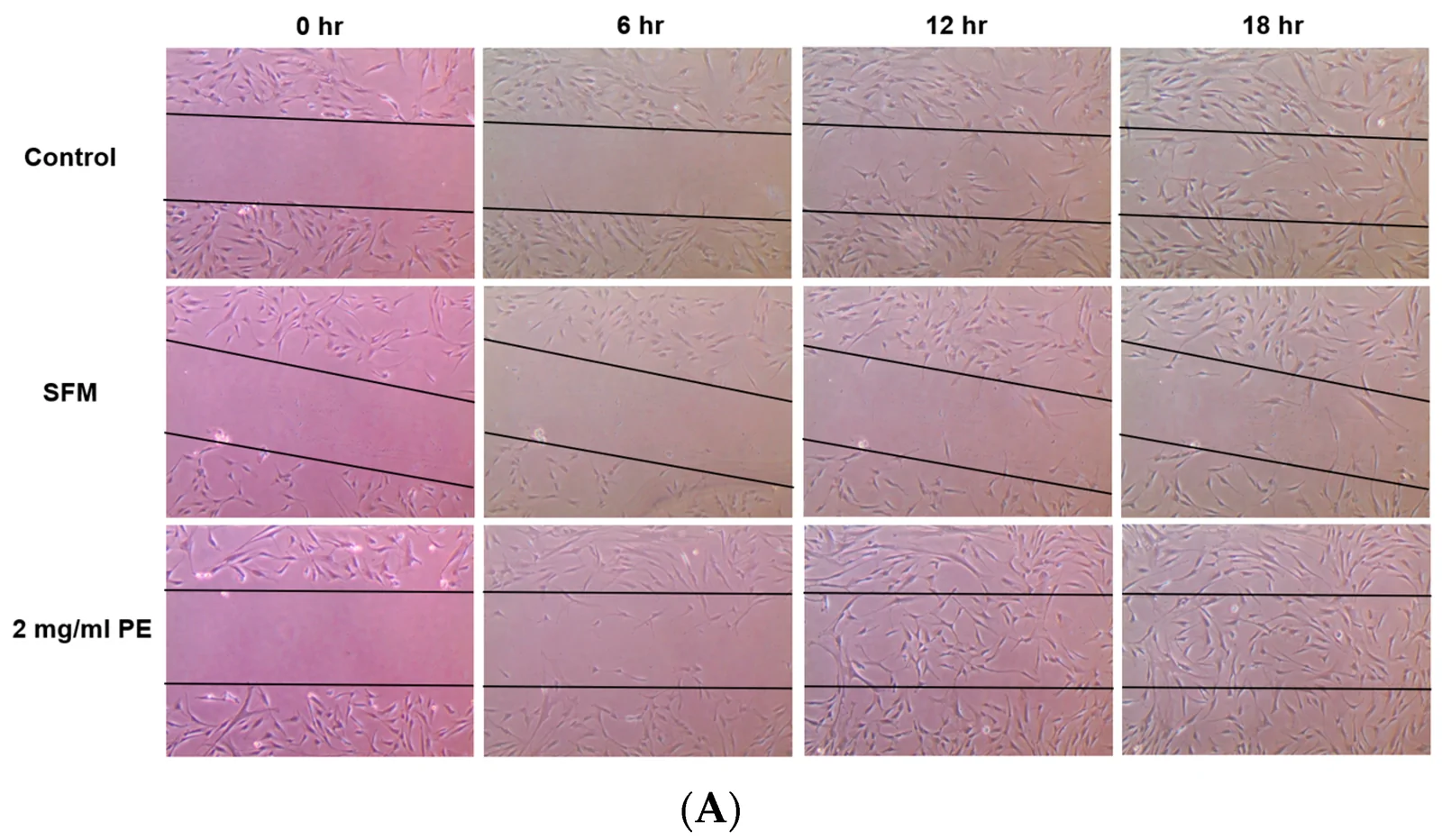

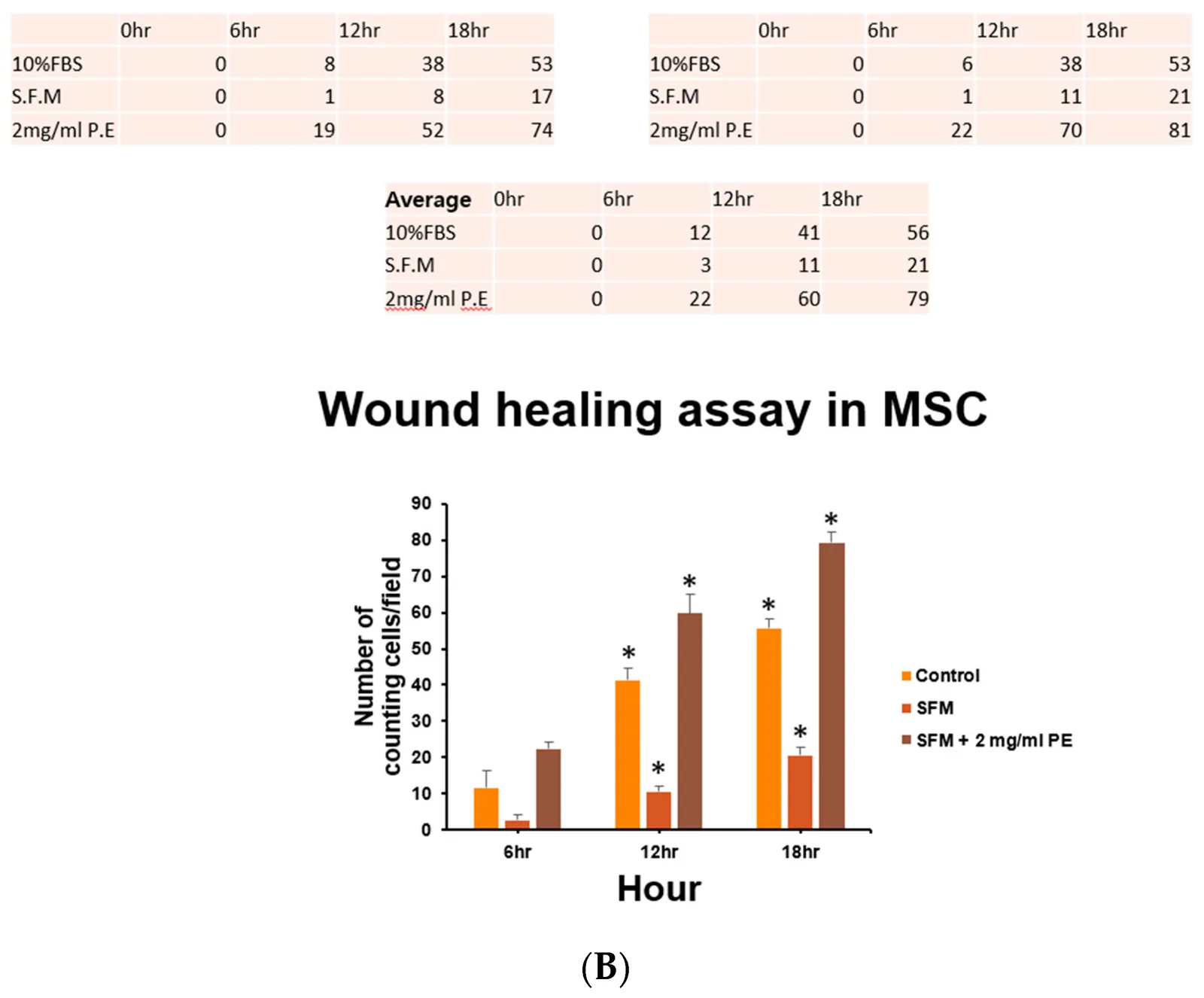
(**A**) **Scratch wound healing assay of MSCs under different treatment conditions**. Representative phase-contrast images showing cell migration at 0, 6, 12, and 18 h after scratch formation in the control, serum-free medium (SFM), and 2 mg/mL placental extract (PE) groups. The PE-treated group demonstrated enhanced wound closure over time compared to the control and SFM groups, indicating increased cell migratory capacity. (**B**) **Quantitative analysis of wound healing assay in MSCs**. Top: Raw and averaged values of wound closure (%) at 0, 6, 12, and 18 h in the 10% FBS (control), serum-free medium (SFM), and 2 mg/mL placental extract (PE) groups. Bottom: Bar graph showing the number of migrated cells per field over time. The PE-treated group demonstrated significantly enhanced cell migration compared to the control and SFM groups, whereas the SFM group showed the lowest migratory activity. Data are presented as mean ± SEM (* *p* < 0.05).

The migratory capacity of MSCs was evaluated using a wound-healing assay over 18 h (Figure 4A,B). In the control group, gradual wound closure was observed over time, with migration rates of 16% at 6 h, 41% at 12 h, and 56% at 18 h. Cells cultured in serum-free medium (SFM) showed minimal migration, with wound closure of 0% at 6 h, 4% at 12 h, and 16% at 18 h, indicating markedly impaired cell motility in the absence of serum factors. In contrast, treatment with placental extract (PE, 2 mg/mL) significantly enhanced MSC migration compared with the control group. Wound closure reached 33% at 6 h, 56% at 12 h, and 74% at 18 h, demonstrating accelerated wound closure and increased cell motility. Overall, these findings indicate that PE markedly promotes MSC migration, whereas serum deprivation suppresses migratory activity.

#### 3.1.3. Placental Extract Reduces Oxidative Stress–Induced Senescence in Mesenchymal Stem Cells

**Figure 5:**
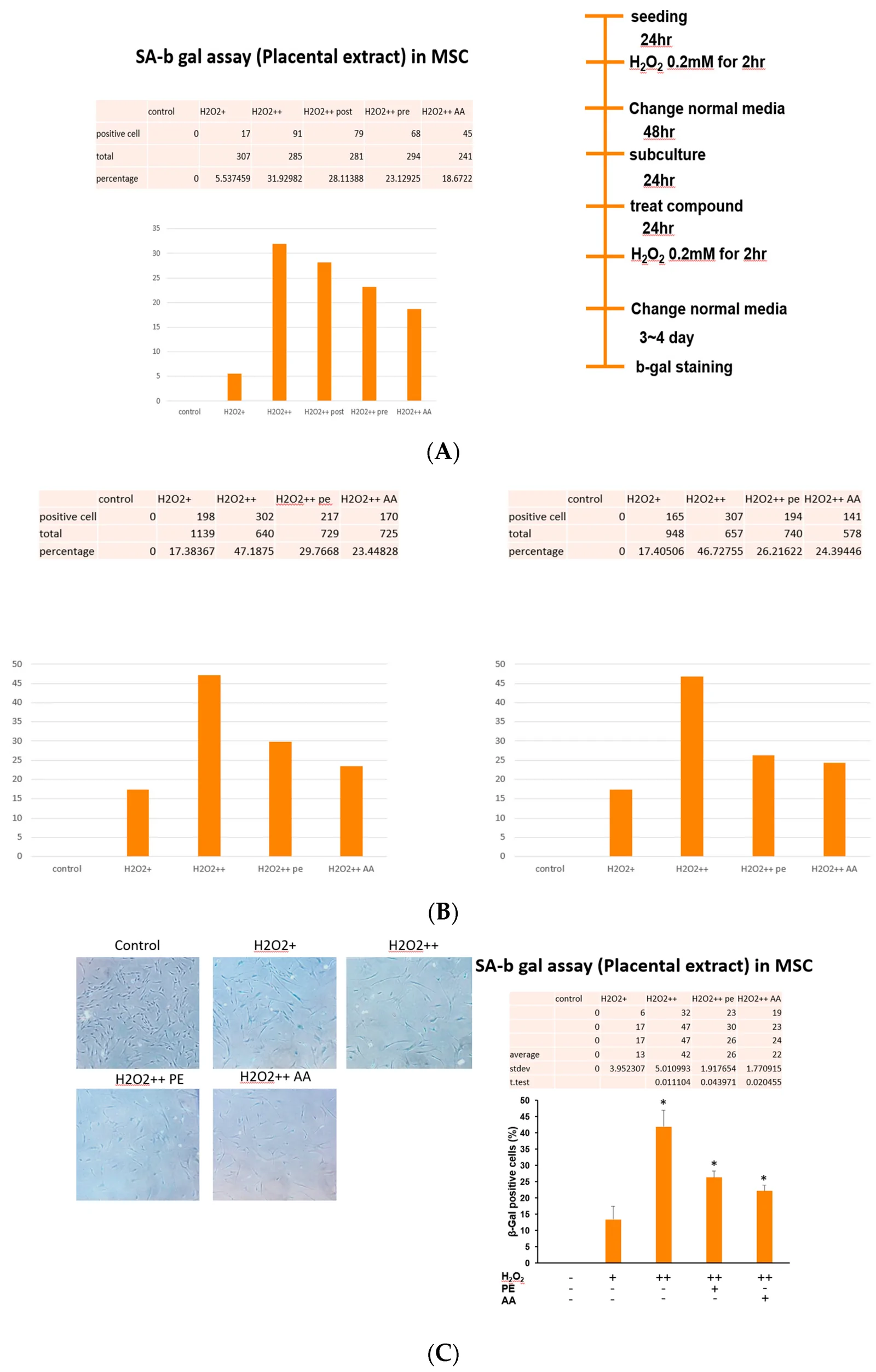
**Senescence-associated β-galactosidase (SA-β-gal) assay in mesenchymal stem cells (MSCs) treated with placental extract under oxidative stress.** (**A**) Quantification of SA-β-gal-positive cells in MSCs under different experimental conditions: control, H_2_O_2_+ (0.2 mM), H_2_O_2_++, H_2_O_2_++ post-treatment, H_2_O_2_++ pre-treatment, and H_2_O_2_++ with ascorbic acid (AA). The percentage of SA-β-gal-positive cells increased markedly following H_2_O_2_ exposure, indicating induction of cellular senescence. Treatment with placental extract (pre- and post-treatment) reduced the proportion of senescent cells compared to the H_2_O_2_++ group, with additional attenuation observed in the AA-treated group. (**B**) Bar graph representing the percentage of SA-β-gal-positive cells across experimental groups. The H_2_O_2_++ group showed the highest level of senescence, while placental extract treatment significantly decreased senescence levels. (**C**) Schematic diagram of the experimental protocol. MSCs were seeded and cultured for 24 h, followed by exposure to H_2_O_2_ (0.2 mM for 2 h) to induce oxidative stress. After medium replacement and subculture, cells were treated with placental extract for 24 h, re-exposed to H_2_O_2_, and then maintained in normal medium for 3–4 days prior to SA-β-gal staining (* *p* < 0.05).

Cellular senescence in MSCs was evaluated using the senescence-associated β-galactosidase (SA-β-gal) assay after oxidative stress induced by H_2_O_2_ (Figure 5A–C). In the control group, no SA-β-gal-positive cells were detected, indicating the absence of basal senescence. Exposure to H_2_O_2_ (0.2 mM) induced a significant increase in senescent cells, with 13 ± 3.95% SA-β-gal-positive cells compared with the control (*p* < 0.05). A higher level of oxidative stress (H_2_O_2_++) further increased the proportion of senescent cells to 42 ± 5.01%, demonstrating a marked induction of cellular senescence. Treatment with PE following oxidative stress significantly reduced the proportion of senescent cells to 26 ± 1.91%, indicating a protective effect against H_2_O_2_-induced senescence (*p* < 0.05 vs. H_2_O_2_++). Similarly, treatment with ascorbic acid (AA) decreased SA-β-gal-positive cells to 24 ± 1.77%, suggesting an antioxidant-mediated reduction in oxidative stress-induced senescence. Overall, these results demonstrate that PE attenuates oxidative stress-induced senescence in MSCs, with effects comparable to those observed with the antioxidant control (AA).

### 3.2. In Vivo Study

#### 3.2.1. Recovery of Vibrissa Fibrillation

**Figure 6:**
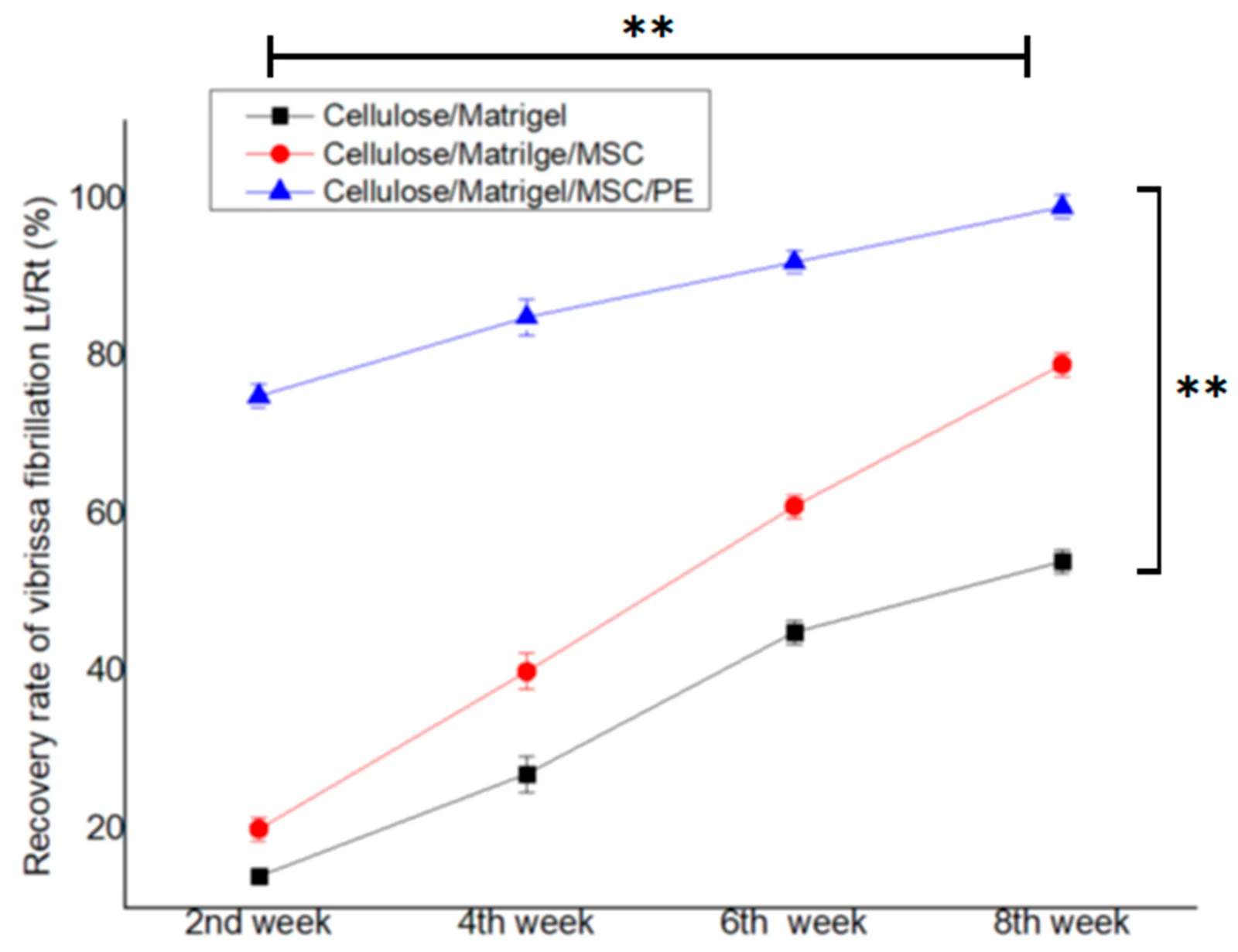
**Recovery of vibrissa fibrillation over time in experimental groups.** Line graph showing the recovery rate of vibrissa fibrillation (Lt/Rt, %) at 2, 4, 6, and 8 weeks in the control, group I, and group II. All groups demonstrated progressive functional recovery over time; however, group II exhibited the highest recovery rates at all time points, followed by group I, while the control group showed the lowest recovery. Data are presented as mean ± SEM. Statistical analysis using repeated-measures ANOVA revealed significant differences among groups over time, with group II showing significantly greater recovery compared to the other groups (** *p* < 0.01).

All rats survived throughout the experimental period, and no postoperative complications were noted. As illustrated in Figure 6, recovery of vibrissa movement was more pronounced in group II compared with the control group. In addition, vibrissa fibrillation progressively improved over time, showing a time-dependent recovery pattern at postoperative weeks 2, 4, 6, and 8 (*p* < 0.05, repeated ANOVA).

#### 3.2.2. Recovery of Threshold of Action Potential

**Figure 7:**
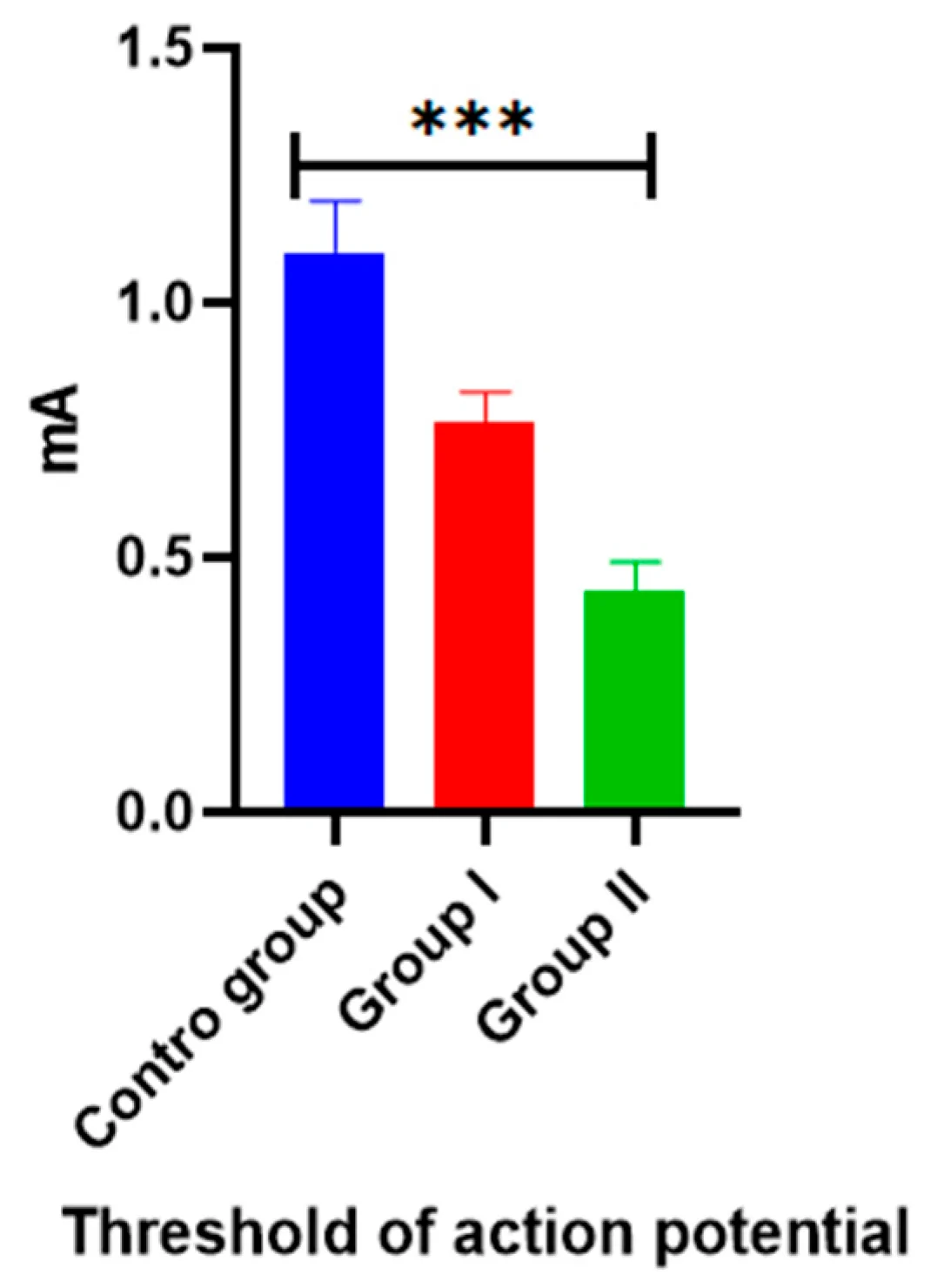
**Threshold of action potential among experimental groups.** Bar graph showing the threshold of action potential (mA) in the control group, group I, and group II. The threshold was highest in the control group and progressively decreased in group I and group II, indicating improved nerve excitability in the treated groups. Data are presented as mean ± SEM. Statistical analysis using one-way ANOVA revealed a significant difference among groups (*p* < 0.0001). Post hoc comparisons demonstrated a significantly lower threshold in group II compared to the control group (*** *p* < 0.001).

**Figure 8:**
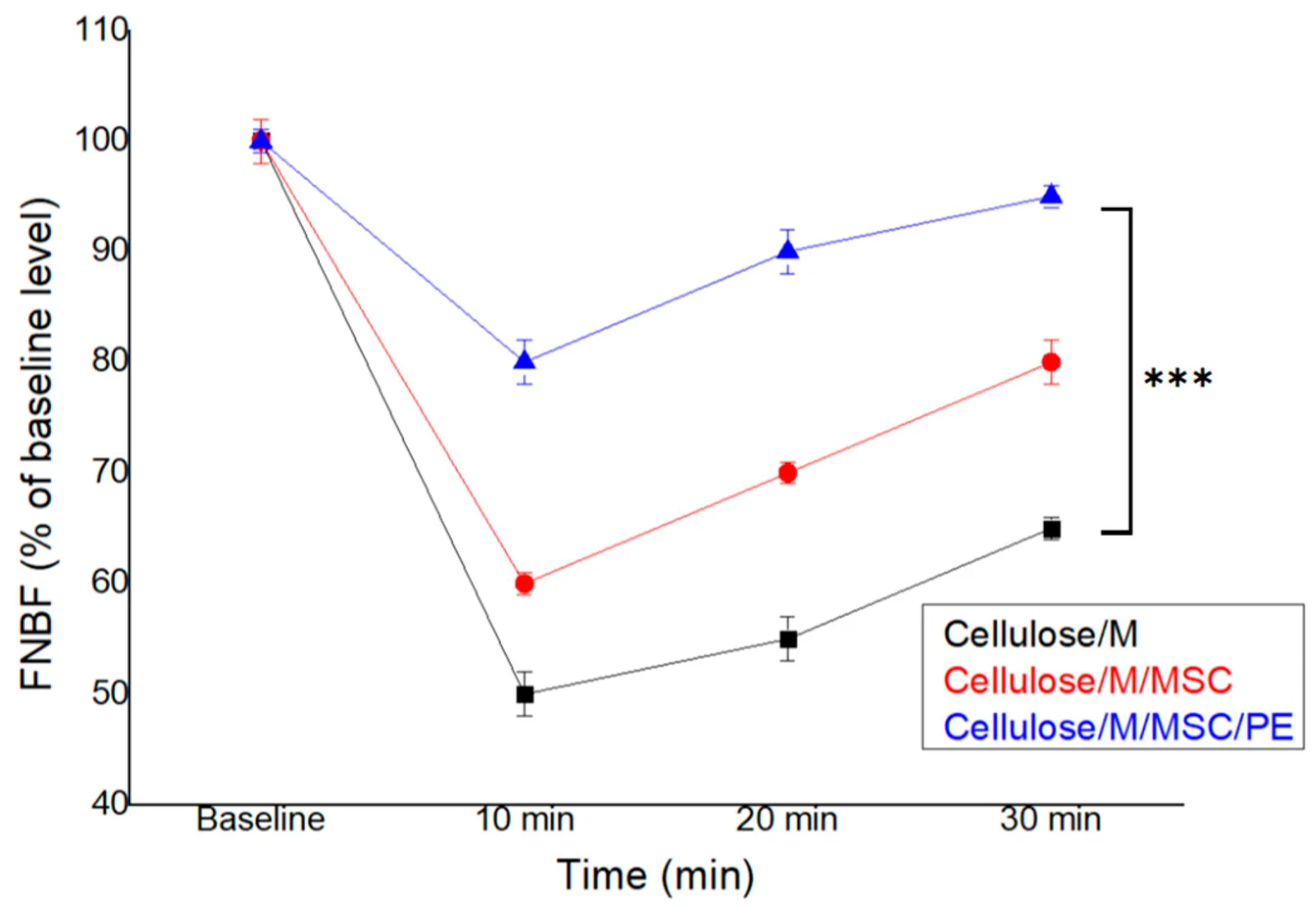
**Changes in facial nerve blood flow (FNBF) measured by laser Doppler flowmetry.** All groups exhibited a marked decrease in FNBF at 10 min, followed by gradual recovery over time. Group II showed significantly greater recovery of FNBF compared to the other groups, particularly at 30 min. Data are presented as mean ± SEM. Statistical analysis demonstrated a significant difference among groups at 30 min (*** *p* < 0.001).

As shown in Figure 7 and Figure 8 weeks after the application of the nerve conduit, group II showed a significantly lower mean threshold of AP than the other two groups. The mean threshold of MAP of group I was significantly lower than that of the control group. 

#### 3.2.3. Facial Nerve Blood Flow (FNBF)

Compared with the sham control group, all treatment groups exhibited significantly improved FNBF. Among them, group II demonstrated the greatest recovery (Figure 8). One-way ANOVA revealed a statistically significant difference (*** *p* < 0.0001). The superior recovery observed in group II is likely due to the combined, synergistic effects of MSC and PE.

#### 3.2.4. Semi-Thin Sections of Regenerated Nerves

**Figure 9:**
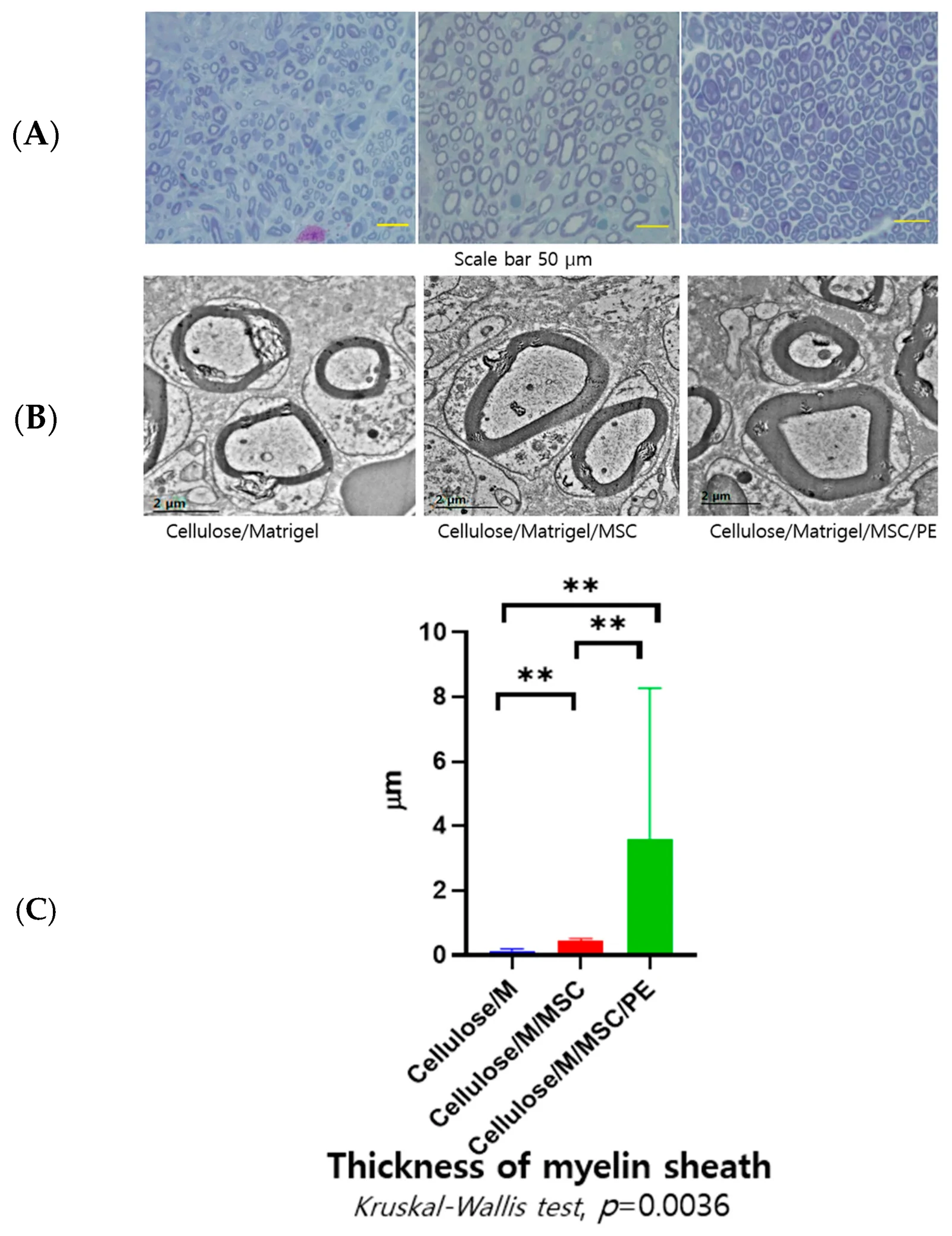
**Histological and ultrastructural evaluation of regenerated nerve fibers in different treatment groups.** (**A**) Light microscopic images (toluidine blue staining) of transverse nerve sections demonstrating myelinated axons in the control, group I, and group II. The group II shows a higher density of uniformly distributed myelinated fibers with more regular morphology compared to the other groups. Scale bar = 50 μm. (**B**) **Transmission electron microscopy (TEM) images of representative nerve fibers from each group.** The control group exhibits relatively thinner myelin sheaths and irregular axonal structures. Group I shows improved myelination and axonal organization. Group II demonstrates thicker, more compact myelin sheaths and well-preserved axonal ultrastructure, indicating enhanced nerve regeneration. Scale bar = 2 μm. (**C**) **Quantitative analysis of myelin sheath thickness in regenerated nerve fibers.** Group II exhibited a significantly greater myelin sheath thickness compared to the other groups, indicating enhanced remyelination. Data are presented as mean ± SEM. Statistical analysis was performed using the Kruskal–Wallis test (*p* = 0.0036), followed by multiple comparisons (** *p* < 0.01).

Toluidine blue staining of semi-thin sections of regenerated nerves showed a stepwise improvement across the three conduit groups at 8 weeks (Figure 9). In the control group, regenerated fibers were relatively sparse, irregularly shaped, and embedded in loose connective tissue. Addition of MSCs (Cellulose/Matrigel/MSC, group I) increased fiber density and produced a more uniform population of small-to-medium myelinated axons. Group II showed the densest and most regular axonal population of the three, with round, well-myelinated fibers more closely resembling normal nerve architecture.

#### 3.2.5. Western Blotting of Facial Muscle

**Figure 10:**
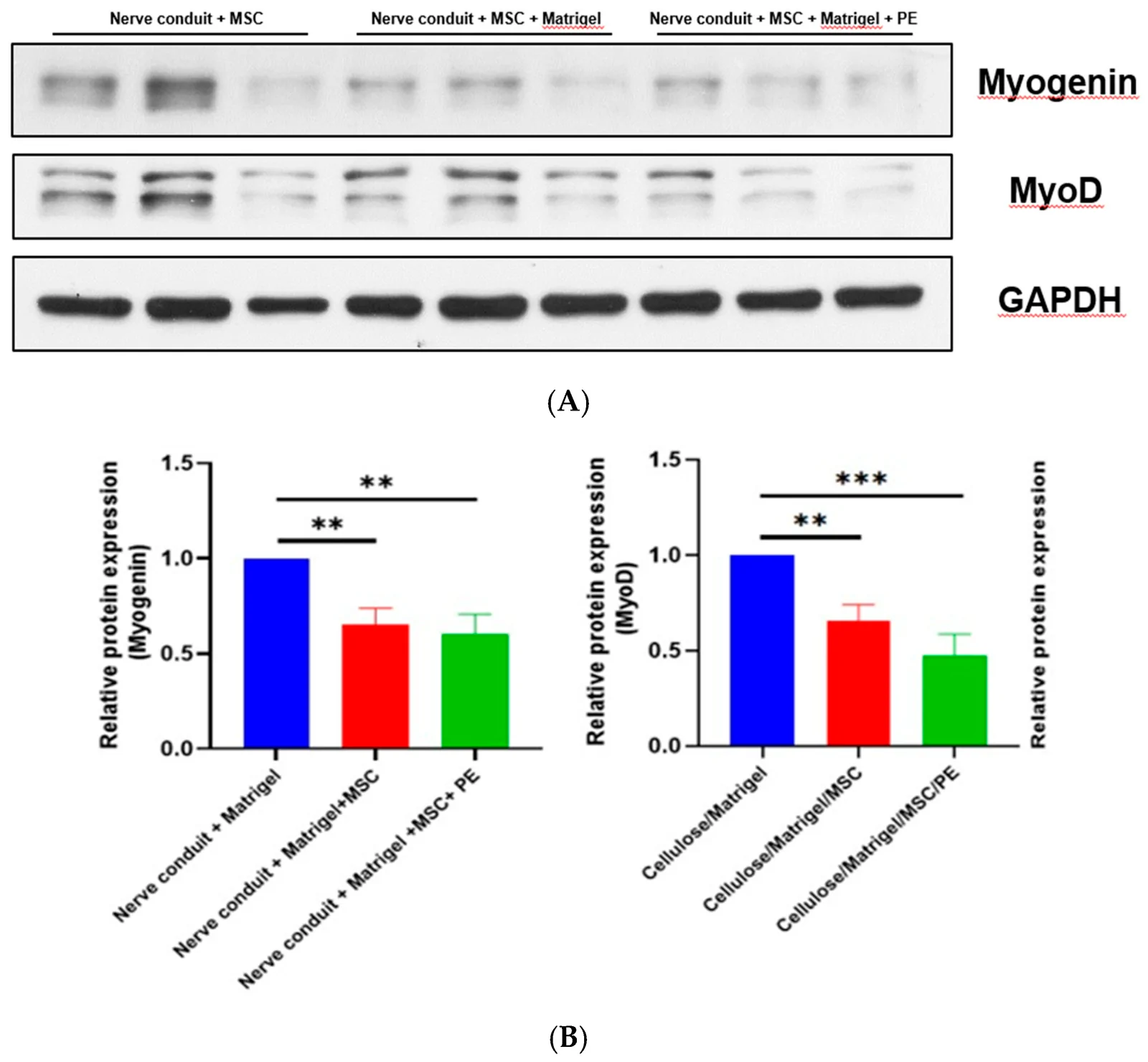
**Western blot analysis of myogenic and neural regeneration-related protein expression in different treatment groups.** (**A**) Representative Western blot bands showing the expression of myogenin (34 kDa) and MyoD (45 kDa) in the Cellulose/Matrigel, Cellulose/Matrigel/MSC, and Cellulose/Matrigel/MSC/PE groups. GAPDH (37 kDa) was used as a loading control. (**B**) Quantitative analysis of relative protein expression normalized to GAPDH. The Cellulose/Matrigel/MSC/PE group demonstrated significantly decreased expression of myogenin and MyoD compared to the other groups, indicating enhanced myogenic differentiation. Data are presented as mean ± SEM. Statistical significance was determined using one-way ANOVA followed by appropriate post hoc testing (** *p* < 0.01, *** *p* < 0.001).

In this study, we evaluated muscle atrophy recovery following a facial nerve transection injury by analyzing the protein expression levels of Myogenin and MyoD in Western blot at the 8-week mark (Figure 10). Three groups were compared. The results revealed that protein levels of Myogenin and MyoD were significantly lower in group II compared to group I. Myogenin is a key regulator of myogenesis and controls muscle atrophy upon denervation. Adult mice lacking myogenin are resistant to muscle atrophy upon denervation [21]. MyoD, like myogenin, is up-regulated in response to denervation [22]. Once regeneration progresses toward maturation, their expression declines; therefore, lower expression at the same postoperative time point may indicate completion of regeneration rather than impaired regeneration [23,24]. The accelerated healing facilitated by Matrigel and PE likely reduces the need for prolonged upregulation of regenerative proteins, further supporting their contribution to a more efficient recovery timeline.

## 4. Discussion

The most prominent finding of this study was that group II demonstrated the total recovery among all experimental groups. This superior outcome suggests a synergistic interaction between PE and MSCs, rather than a simple additive effect. MSCs are well recognized for their role in peripheral nerve regeneration, primarily through paracrine mechanisms rather than direct neuronal differentiation [25,26,27,28]. MSCs secrete a wide range of neurotrophic factors, including nerve growth factor, brain-derived neurotrophic factor, and glial cell line-derived neurotrophic factor, which promote axonal elongation and Schwann cell activation. However, the regenerative capacity of MSCs is highly dependent on the surrounding microenvironment, and their survival and functional activity may be limited in injured or ischemic nerve tissue.

Placental extract appears to play a critical role in optimizing this regenerative microenvironment. It contains multiple bioactive components, such as epidermal growth factor, fibroblast growth factor, vascular endothelial growth factor, and anti-inflammatory cytokines [14,15]. These factors are known to enhance angiogenesis, suppress excessive inflammation, and improve tissue perfusion, all of which are essential for effective nerve regeneration. In the present study, the significantly increased facial nerve blood flow observed in the placental extract-treated group supports this angiogenic and microcirculatory enhancement. The enhanced angiogenesis by PE was observed by facial nerve blood flow by laser Doppler blood flowmeter in Figure 9. Importantly, the combination of placental extract with MSCs resulted in markedly superior functional recovery, electrophysiological responses, and ultrastructural nerve maturation compared with MSC treatment alone. This suggests that placental extract not only provides independent regenerative cues but also potentiates the therapeutic efficacy of MSCs by enhancing their survival, paracrine activity, and interaction with host Schwann cells. The improved axonal density, thicker myelin sheath, and more normalized g-ratio observed on transmission electron microscopy further indicate that this synergistic effect extends beyond early regeneration to promote long-term nerve maturation.

From a tissue engineering perspective, these findings highlight the importance of multi-component regenerative strategies that integrate structural scaffolds, cellular therapy, and biochemical modulation. While cellulose-based nerve conduits provide essential physical guidance and Matrigel offers extracellular matrix support, the addition of MSCs and placental extract appears to recreate a more physiologically relevant regenerative niche. This integrated approach addresses not only axonal guidance but also vascularization, cellular survival, and microenvironmental regulation.

Clinically, the observed synergy between placental extract and MSCs suggests a promising therapeutic avenue for facial nerve reconstruction, particularly in situations where autologous nerve grafting is limited or undesirable. Future studies should investigate dose optimization, long-gap nerve defects, and long-term functional outcomes to further validate the translational potential of this combined regenerative strategy.

In this study, we evaluated muscle atrophy recovery following a facial nerve transection injury by analyzing the protein expression levels of Myogenin and MyoD using Western blot at the 10-week mark. Three experimental groups were compared. The results revealed that protein levels of Myogenin and MyoD were significantly lower in the Matrigel and Matrigel + PE groups compared to the MSC-only group.

This reduction in protein expression in the Matrigel and Matrigel + PE groups suggests that these groups may have reached a more advanced stage of recovery compared to the MSC-only group. The elevated protein levels observed in the MSC-only group indicate ongoing regenerative processes, whereas the Matrigel and PE groups appear to have completed much of the recovery by this time point. Therefore, the lower expression levels of these regeneration markers in the Matrigel-containing groups likely reflect accelerated recovery progression, highlighting the role of Matrigel and PE in promoting a faster return to a stable, post-recovery state.

These findings are consistent with previous studies demonstrating that Matrigel and PE, in conjunction with MSCs, enhance muscle and nerve repair through mechanisms such as supporting angiogenesis, reducing fibrosis, and accelerating synaptic reorganization [24,28]. The accelerated healing facilitated by Matrigel and PE likely reduces the need for prolonged upregulation of regenerative proteins, further supporting their contribution to a more efficient recovery timeline.

Our findings are consistent with the emerging paradigm in peripheral nerve tissue engineering that multi-component regenerative strategies outperform single-agent approaches. A recent comprehensive review by Wei et al. emphasized that MSCs promote peripheral nerve regeneration not only through paracrine secretion of neurotrophic factors but also through intercellular mitochondrial transfer via tunneling nanotubes and the release of exosomes containing regulatory microRNAs, both of which enhance Schwann cell proliferation and axonal regrowth [29]. The multi-modal mechanism of MSC action observed in our study—enhanced axonal density, improved myelination, and accelerated functional recovery—aligns with these findings and supports the notion that the regenerative microenvironment created by MSCs is amplified when supplemented with biologically active agents such as placental extract.

Recent advances in nerve guidance conduit design further contextualize our approach. Yang et al. provided a comprehensive overview of biomaterial-based tissue engineering strategies for peripheral nerve injury repair, highlighting that the integration of bioactive fillers within the conduit lumen—including hydrogels, neurotrophic factors, and stem cells—substantially improves regeneration outcomes compared with hollow conduits alone [30]. Our group II construct exemplifies this strategy by combining structural guidance with matricellular support, cellular therapy, and a rich cocktail of endogenous growth factors. Similarly, Fakhr et al. underscored the importance of extracellular matrix proteins, topographical cues, and biochemical signals in creating a permissive regenerative microenvironment within nerve conduits [31]. The Matrigel component in our construct serves precisely this role by providing laminin- and collagen IV-rich basement membrane cues that enhance MSC adhesion, survival, and neurotrophic factor secretion.

The biological rationale for incorporating placental extract is further strengthened by Alimu et al., who reviewed the diverse bioactive composition of human placental extracts and reported that peptides derived from placental tissue enhance the expression of brain-derived neurotrophic factor (BDNF) and nerve growth factor (NGF) in cellular models, supporting neuronal growth and maintenance [32]. Recent studies on facial nerve regeneration using alternative cellular approaches also provide a useful benchmark for our findings.

## 5. Limitations

Several limitations of this study should be acknowledged. First, the nerve gap created in this study was relatively short (2 mm), which may not fully reflect the challenges encountered in clinical scenarios involving longer nerve defects. It is difficult to create a large gap of 2 mm or more in the main trunk of the facial nerve. Therefore, to create a large gap, a 5 mm gap in the buccal or mandibular branch is possible [33]. However, when the main trunk is transected, it is easier to observe functional recovery because all branches are paralyzed. Second, this study evaluated functional recovery primarily by vibrissa movement without assessment of eye closure or blink function. Although whisker movement is a sensitive and well-established behavioral outcome in rodent facial nerve regeneration models, recovery of orbicularis oculi function has greater clinical relevance because incomplete eye closure is a major manifestation of facial paralysis in humans. Future studies should incorporate standardized measurements of eye closure, blink reflex, or eyelid function in addition to vibrissa analysis to provide a more comprehensive assessment of facial nerve recovery and improve the translational value of the experimental findings.

Third, the follow-up period was limited to 8 weeks, and while time-dependent improvements were observed, longer-term functional outcomes, including assessment of potential aberrant reinnervation or synkinesis, were not evaluated. Fourth, while the Western blot analysis provided molecular evidence of regeneration, quantitative assessment of neurotrophic factor expression within the conduit microenvironment and at the injury site was not performed. Fifth, the optimal dose and concentration of placental extract were not systematically investigated in vivo; although in vitro viability data guided the selected concentration, dose–response studies in the animal model would strengthen the translational relevance of our findings. Sixth, this study used a rat model, and interspecies differences in nerve anatomy, immune response, and regeneration capacity may limit the direct translation of these results to human clinical applications. Finally, the precise molecular mechanisms underlying the synergistic interaction between MSCs and placental extract remain to be elucidated; future studies incorporating transcriptomic or proteomic analyses at different time points would help clarify the signaling pathways involved.

## 6. Conclusions

This study demonstrates that a cellulose-based nerve conduit loaded with Matrigel, mesenchymal stem cells, and human placental extract synergistically enhances the regeneration of a transected rat facial nerve. The in vitro experiments confirmed that placental extract promotes MSC migration and attenuates oxidative stress–induced senescence without compromising cell viability at the selected concentration. In the in vivo facial nerve gap model, the group II construct yielded significantly superior outcomes across all evaluation parameters, including vibrissa movement recovery, action potential threshold, facial nerve blood flow, axonal density, myelin sheath thickness, and expression of myogenic and neural regeneration markers, compared with the conduit containing MSCs alone or Matrigel only. These findings indicate that placental extract optimizes the regenerative microenvironment by providing a broad spectrum of endogenous growth factors and anti-inflammatory mediators that potentiate the paracrine and trophic activities of MSCs. The multi-component tissue engineering strategy employed herein—combining structural guidance, extracellular matrix support, cellular therapy, and biochemical modulation within a single construct—represents a promising approach for peripheral facial nerve reconstruction. Future investigations should focus on longer nerve gap models in the sciatic nerve, extended follow-up periods, dose optimization of placental extract, and elucidation of the molecular signaling pathways underlying the MSC–PE synergy to advance the translational potential of this regenerative platform toward clinical application.

## Data Availability

The original contributions presented in this study are included in the article. Further inquiries can be directed to the corresponding author.
